# Supplementary material for: How to improve patient safety in fragile, conflict-affected and vulnerable settings: a Delphi study protocol
Source: BMJ Open. 2021 Oct 6;11(10):e052960. doi: 10.1136/bmjopen-2021-052960 (PMC8496379; doi:10.1136/bmjopen-2021-052960)
Supplement: Supplementary data [file bmjopen-2021-052960supp001.pdf]

Appendix

**Select Delphi study limitations**	**Reflections on limitations in the FCV research content**	**Mitigation strategies in the FCV research context**
Challenge in defining consensus	No unique challenges in defining consensus in FCV research.	A clear plan to define consensus, based on best practice and in line with published research, agreed by the research team could be beneficial at the protocol development stage.
High attrition rates	Attrition rates in FCV research are likely to be higher than in other areas. Individuals involved in FCV research working in FCV settings will likely have several competing priorities due to a lack of healthcare resources in these settings.	Attrition rates should be considered in protocol development, with steps taken to reduce the number of rounds and length of questionnaires to capture only the information required for the study.
Anonymity and accountability	Anonymity may play a more complex role in FCV research as the information discussed by participants is likely to have an emotional component and may involve the sharing of negative experiences.	The research team should be mindful of the negative aspects of anonymity in Delphi studies and work with participants to ensure that the sharing of personal experiences by participants (and respective analysis) are undertaken in a respectful manner with particular attention to the emotive nature of the topic, and participant well-being.

Internet access	Internet access challenges for some participants in FCV settings is likely to be a challenge. In FCVs, connectivity may be poor or non-existent, thus excluding individuals in these settings from participating in the study.	Depending on the nature of the research project and funding available there are several mitigation strategies that could be employed. For example, Donohoe et al. (2012) suggest making paper surveys available to participants with no internet access / limited access or offering financial assistance to facilitate access where appropriate [1].

Loss of research control	In FCV settings, there are additional risks in losing research control, specifically missing deadlines due to a range of possible distractions and delays to participation. Additionally, the security of data related to individual participation may be more challenging in the FCV environment.	The research team could develop a risk register outlining risks to maintaining control. Additionally, Donohoe et al. (2012) note the value of identifying possible distractions/delays to participation [1]. In the FCV setting, FCV-based disruptions (e.g., political challenges, weather challenges) should also be considered.<br>Similarly, Donohoe et al. (2012) recommend ensuring secure survey access through unique passwords and hyperlinks [1]. In FCV settings, this may be increasingly important as systems may have lower cybersecurity protection in place and healthcare organisations may be a frequent the target of attacks (both physical and cyber) [2,3].

References

1. Donohoe H, Stellefson M, Tennant B. Advantages and Limitations of the e-Delphi Technique, American Journal of Health Education. 2013; (1): 38-46.
2. International Committee of the Red Cross. What limits does the law of war impose on cyber attacks? Available from: https://www.icrc.org/en/doc/resources/documents/faq/130628-cyber-warfare-q-and-a-eng.htm [cited 2021 April 21].

3. International Committee of the Red Cross. Call to governments: Work together to stop cyber attacks on health care. Available from: https://www.icrc.org/en/document/governments-work-together-stop-cyber-attacks-health-care [cited 2021 April 21].
