# Supplementary material for: How to improve patient safety in fragile, conflict-affected and vulnerable settings: a Delphi study protocol
Source: BMJ Open. 2021 Oct 6;11(10):e052960. doi: 10.1136/bmjopen-2021-052960 (PMC8496379; doi:10.1136/bmjopen-2021-052960)
Supplement: Author's manuscript [file bmjopen-2021-052960.draft_revisions.pdf]

BMJ Open

BMJ Open is committed to open peer review. As part of this commitment we make the peer review history of every article we publish publicly available.

When an article is published we post the peer reviewers' comments and the authors' responses online. We also post the versions of the paper that were used during peer review. These are the versions that the peer review comments apply to.

The versions of the paper that follow are the versions that were submitted during the peer review process. They are not the versions of record or the final published versions. They should not be cited or distributed as the published version of this manuscript.

BMJ Open is an open access journal and the full, final, typeset and author-corrected version of record of the manuscript is available on our site with no access controls, subscription charges or pay-per-view fees (http://bmjopen.bmj.com).

If you have any questions on BMJ Open's open peer review process please email info.bmjopen@bmj.com

BMJ Open

How to improve patient safety in fragile, conflict-affected and vulnerable settings: a Delphi study protocol

Journal:	*BMJ Open*
Manuscript ID	bmjopen-2021-052960
Article Type:	Protocol
Date Submitted by the Author:	29-Apr-2021
Complete List of Authors:	Shaw, Alexandra ; Imperial College London, Patient Safety Translational Research Centre<br>O'Brien, Niki; Imperial College London, Institute of Global Health Innovation<br>Flott, Kelsey; Imperial College London,<br>Leatherman, Sheila; University of North Carolina at Chapel Hill Gillings School of Global Public Health<br>Durkin, Michael; Imperial College London, Institute for Global Health Innovation; Imperial College London, Centre for Health Policy, Institute of Global Health Innovation<br>Darzi, Ara; Imperial College London, Institute of Global Health Innovation<br>Neves, Ana Luisa; Imperial College London,
Keywords:	HEALTH SERVICES ADMINISTRATION & MANAGEMENT, Protocols & guidelines < HEALTH SERVICES ADMINISTRATION & MANAGEMENT, Organisation of health services < HEALTH SERVICES ADMINISTRATION & MANAGEMENT, International health services < HEALTH SERVICES ADMINISTRATION & MANAGEMENT, Quality in health care < HEALTH SERVICES ADMINISTRATION & MANAGEMENT, QUALITATIVE RESEARCH

SCHOLARONE™
Manuscripts

I, the Submitting Author has the right to grant and does grant on behalf of all authors of the Work (as defined in the below author licence), an exclusive licence and/or a non-exclusive licence for contributions from authors who are: i) UK Crown employees; ii) where BMJ has agreed a CC-BY licence shall apply, and/or iii) in accordance with the terms applicable for US Federal Government officers or employees acting as part of their official duties; on a worldwide, perpetual, irrevocable, royalty-free basis to BMJ Publishing Group Ltd ("BMJ") its licensees and where the relevant Journal is co-owned by BMJ to the co-owners of the Journal, to publish the Work in this journal and any other BMJ products and to exploit all rights, as set out in our licence.

The Submitting Author accepts and understands that any supply made under these terms is made by BMJ to the Submitting Author unless you are acting as an employee on behalf of your employer or a postgraduate student of an affiliated institution which is paying any applicable article publishing charge ("APC") for Open Access articles. Where the Submitting Author wishes to make the Work available on an Open Access basis (and intends to pay the relevant APC), the terms of reuse of such Open Access shall be governed by a Creative Commons licence – details of these licences and which Creative Commons licence will apply to this Work are set out in our licence referred to above.

Other than as permitted in any relevant BMJ Author's Self Archiving Policies, I confirm this Work has not been accepted for publication elsewhere, is not being considered for publication elsewhere and does not duplicate material already published. I confirm all authors consent to publication of this Work and authorise the granting of this licence.

For peer review only

How to improve patient safety in fragile, conflict-affected and vulnerable settings: a Delphi study protocol

Alexandra Shaw[1], Niki O'Brien[1], Kelsey Flott[1], Sheila Leatherman[3], Mike Durkin[1], Ara Darzi[1], Ana Luisa Neves[1,2]

[1] Imperial NIHR Patient Safety Translational Research Centre, Institute of Global Health Innovation, Department of Surgery and Cancer, Imperial College London, London, UK

[2] Department of Community Medicine, Information and Health Decision Sciences (MEDCIDS) / Center for Health Technology and Services Research (CINTESIS), University of Porto, Porto, Portugal.

[3] UNC Gillings School of Global Public Health, University of North Carolina

Corresponding author

Niki O'Brien

Institute of Global Health Innovation,

QEQM Wing, St Mary's Campus,

London W2 1NY

n.obrien@imperial.ac.uk

Keywords: Patient Safety, fragile, conflict-affected and vulnerable settings; FCVs; organisation of health services; international health services; quality in healthcare; qualitative research; research methods; protocol; Delphi study.

Word count: 3198

Patient and public involvement statement: It was not appropriate or possible to involve patients or the public in the design, or conduct, or reporting, or dissemination plans of our research as this is a study protocol for determining patient safety priorities.

For peer review only - http://bmjopen.bmj.com/site/about/guidelines.xhtml

Abstract

Introduction: There is a high burden of adverse events and poor outcomes in fragile, conflict-affected, and vulnerable (FCV) settings. Thus far, there has been limited research on the improvement of patient safety and implementation of interventions in FCV settings.

Objective: This study intends to generate a consensus on the most relevant and applicable patient safety interventions for these settings from front line clinicians, managers/ administrators, NGOs, policy makers, and researchers, and gather information on the enabling factors and barriers for their effective implementation.

Methods and Analysis: The study utilises a Delphi research approach, specifically an eDelphi. This protocol outlines research that will be conducted online with expert participants from a range of backgrounds, including those working in a variety of FCV settings. Participants will be established contacts known to the research team or recruited via snowballing sampling. Research will be conducted across four to five rounds, with each round taking approximately 15 minutes to complete. Consensus will be considered reached based on a defined minimum mean score and either an interquartile range or a percentage of participants who gave the set minimum mean score. Participants will also complete a free text questionnaire on the strengths, weaknesses, opportunities, and threats (SWOT) to the implementation of patient safety interventions in FCVs. The Consolidated Criteria for Reporting Qualitative studies will be used to ensure the study meets the recommended standards of qualitative data reporting (COREQ criteria). Data analysis will be completed on Microsoft Excel and NVivo. The primary outcome of the study will be a list of the most relevant and applicable patient safety interventions for FCV settings, and the enabling factors and barriers associated.

Ethics and dissemination: The study has received approval from Imperial College London Ethics Committee. Anonymous results will be made available to the public, academic organisations, and policymakers.

For peer review only

Strengths and limitations of this study

- This study will seek to understand what interventions could help to improve patient safety from frontline clinicians, managers/administrators, researchers and policy makers with significant experience in FCV settings.
- A key strength of this study is the utilisation of an eDelphi approach offering flexibility and anonymity to facilitate more open discussion and the ability to conduct research in the COVID-19 era without transmission risk for participants.
- This study has the potential to start to build an evidence base for which interventions could be developed further and evaluation undertaken.
- This study has the potential to draw attention and funding to this area and contribute to policy and real-world interventions to improve the safety of care in FCV settings.
- Limitations of the study include the potential exclusion of those working in the field due to unreliable or lack of internet access, and higher attrition rates among participants due to the nature of the work and conflicting priorities, which we will attempt to mitigate with careful consideration of the participant list and having as few rounds and short length of survey as possible.

For peer review only - http://bmjopen.bmj.com/site/about/guidelines.xhtml

Introduction

The safety of patients is a critical element of well-functioning health systems globally and is an essential component of improving health outcomes and achieving universal health coverage (UHC). Whilst the focus of health system strengthening has historically been on UHC, there has been increasing acknowledgement in recent years that without improving safety and a goal to achieve high quality care, healthcare will not provide the desired outcomes. In low- and middle-income countries (LMICs) attention to patient safety is particularly important given the prevalence of patient safety incidents in these settings; accounting for an estimated 134 million adverse events, and 2.5 million deaths annually [1]. While the need to improve patient safety is an increasingly recognised priority within the global health agenda, there is still much to be done to develop and implement appropriate tools to achieve this goal [2].

The term fragile, conflict-affected and vulnerable (FCV) settings describes any setting of crisis, and may include armed conflict, complex emergencies, natural disasters and disruption to public services [3]. At present there is no widely accepted and precise global definition of FCV settings and the term may be used interchangeably with terms such as 'settings of extreme adversity', 'humanitarian settings' and 'acute', 'protracted' or 'complex' emergencies [4]. Approximately 2 billion people are currently estimated to live in FCV settings with this figure set to rise [5]. Estimates suggest large numbers of preventable deaths take place in FCV settings including; 60% of preventable maternal deaths, 53% of deaths in children under five years, and 45% of neonatal deaths [4].

To reduce the impact of unsafe care on patient outcomes in FCV settings, patient safety interventions could be implemented to reduce incidents and improve the standard of care. Incidents of unsafe care range from wrong site surgery, to incorrect patient identification and delayed or missed opportunities for intervention resulting in preventable morbidity and mortality [4]. Potential interventions cover a range of areas including, for example, infection prevention and control, health worker training and point of care, however barriers to implementation can be considerable in these settings. An example of a specific intervention is the WHO Surgical Safety Checklist which, in a study by Haynes et al. (2009), the rate of death before and after the checklist was introduced declined from 1.5% to 0.8% and inpatient complications dropped from 11% to 7%. [6,7].

For peer review only - http://bmjopen.bmj.com/site/about/guidelines.xhtml

Despite the significant impact of patient safety incidents in FCV settings, there is limited evidence about the most applicable patient safety interventions, and how best to support these communities to improve the safety of care. Such a gap in knowledge makes it challenging to assess the quality and safety of existing interventions, measure their accessibility and effectiveness, address any context specific challenges, and develop new interventions [4]. In other healthcare settings it is possible to capture data and record it over time, however in FCV settings this can be challenging due to system, local or organisational leadership instability, lack of technology and infrastructure and clinical priorities due to the demand for care on limited resources and staff. As such, a critical starting point must be to build an evidence base and identify which interventions are considered most useful and relevant in FCV settings and furthermore what is required to develop such interventions to be accessible and effective. It is unlikely that this evidence will initially come from traditional academic sources, particularly with the exacerbation of fragility caused by the COVID-19 pandemic, however there is an opportunity to bring together and build on the knowledge held in the experiences of experts and practitioners working in these contexts.

The aim of the study is to identify the most relevant patient safety interventions for these settings and also gather information on the enabling factors and barriers for their implementation. The Delphi study design used by the research team sought to gain consensus on the question 'What is needed to improve patient safety in FCV settings?' from clinicians working on the ground, managers/ administrators, policy makers, researchers and non-governmental organizations (NGOs).

Methods and analysis

Study design

The study will use a Delphi methodology to seek consensus on a question among a group of experts. Where there is a lack of research evidence and a desire to reach consensus, a commonly used formal consensus method is the Delphi technique, which uses rounds of questionnaires to collect data and achieve group consensus [8]. As the research will be conducted remotely online, through this paper we utilise both the term "Delphi", to describe the established research technique, and the term "eDelphi" to describe our proposed research in the FCV setting.

For peer review only - http://bmjopen.bmj.com/site/about/guidelines.xhtml

Recruitment

Delphi studies employ a panel of expert members, as opposed to a random representative sample. In this study, 'expert' will be defined as a professional with experience working in health delivery and/or patient safety in FCV settings. Additional inclusion criteria is being aged over the 18 and being able to understand English.

Purposive sampling will be used to ensure representativeness from a variety of roles (clinicians, managers/administrators, policymakers, researchers, and NGOs) at the organisational and governmental level. The majority of participants will be established contacts known to the research team via the Institute of Global Health Innovation's (IGHI) Leading Health Systems Network (LHSN) and the NIHR Imperial Patient Safety Translational Research Centre (PSTRC). Other relevant participants will be identified through the team's contacts at the World Health Organization (WHO) or using snowballing sampling. The use of snowballing sampling is needed due to this being a diverse field, with no centralised community of practice across FCV settings. Both IGHI and Sheila Leatherman have world renowned previous research and a wide range of possibilities to access contacts in these areas. Attention will be paid to ensure the range of participants is geographically diverse and reflects the wide range of FCV settings, including settings of current or recent armed conflict, complex emergencies, natural disasters and disruption to public services.

There is no agreement in the literature on the optimal number of participants to include in a Delphi survey [9]. Therefore, we will include all 'experts' who express an initial interest to participate. An initial invitation email will be sent to possible participants identified by the research team. Participants will be made aware that their participation is voluntary and if they do not take part in the study it will not affect their relationship with the study team. Each participant will be given a Participant Information Sheet and provided with the opportunity to address any queries they may have with a researcher.

Data collection and analysis

For peer review only - http://bmjopen.bmj.com/site/about/guidelines.xhtml

The eDelphi study will seek to gain consensus on the question 'What is needed to improve patient safety in FCV settings?'. Qualtrics will be used to develop each round of the online questionnaire survey. The survey will be conducted across four to five rounds and each round of the survey will take approximately 15 minutes or less to complete. A link to each survey will be distributed via email to all participants followed by two reminder emails, at one week intervals, per survey round.

In Round 1, experts will be asked to list as many interventions as they want or a set number of responses to the question: 'What is needed in order to improve patient safety in FCV settings?'. The data will be analysed by two independent qualitative researchers using thematic analysis [10]. This process will be supported by the use of NVivo 10 qualitative data analysis software package. To cross-check data analysis and ensure data quality, consistency in approach and transparency of analytical decision making, 50% of the data will be read and coded independently by a second experienced qualitative researcher. Differences in interpretation will be resolved through discussion between the two researchers and the wider research team. A number of statements representing each theme will then be developed.

In Rounds 2-3, experts will be asked to rank the thematic areas on a Likert scale (1-10). Participants will also be asked to determine the accuracy of the thematic areas of patient safety interventions outlined on a Likert scale (1-10). Consensus will be considered based on the minimum mean score and either an interquartile range or a percentage of participants who gave the set minimum mean score. The responses will be subsequently analysed and interventions without consensus will be included in the subsequent round.

Finally, in Round 4, experts will be asked to order each of the interventions that received consensus in Rounds 2 and 3 based on which are likely to be most impactful. Responses will be subsequently analysed and ultimately mapped in thematic areas ranked by importance.

Additionally, experts will be asked to complete a free text section based on the strengths, weaknesses opportunities and threats (SWOT) to the implementation of patient safety interventions in FCV settings. A total of 2 independent researchers systematically will review the free-text answers, using the framework analysis method, which includes 5 main stages: familiarisation, identifying a thematic framework, indexing, charting, and mapping, and interpretation [11]. At every stage of the data analysis process, the coding framework will be kept

For peer review only - http://bmjopen.bmj.com/site/about/guidelines.xhtml

deductive and inductive, allowing the ongoing inclusion of new themes. Identified themes will be supported by quotations from the free-text answers. The Consolidated Criteria for Reporting Qualitative studies will be used to ensure the study meets the recommended standards of qualitative data reporting (COREQ criteria) [12].

The timeline for data collection, analysis and report preparation will be approximately 6-9 months. This indicative timeline reflects the need to be flexible to achieve a minimum level of participation in each round of the Delphi study, but also provide deadlines for participants to encourage continued engagement and accountability.

Patient and public involvement

Patient partners will be included in the interpretation of our results, in the co-development of a dissemination strategy, and in summarising the research findings into lay summaries and reports, in order to raise awareness and stimulate public participation on this topic. The research team at the Imperial NIHR PSTRC has a dedicated PPIE lead and a Research Partners Group.

Ethics and dissemination

This project has been submitted to the Imperial College London Ethics Committee, reference number 20IC665. Consent has been incorporated into the survey alongside the participant information provided.

The results of this study will be published in a peer-reviewed journal and the intention is that this will begin to build an evidence base for further research and provide background for future grant applications. It would also be hoped that the findings of this study will be disseminated via presentation to international audiences through conferences. It is intended that this research will inform future translational research and also provide a starting point to develop in-country partnership to increase capacity building for research and patient safety intervention implementation in these settings.

Discussion

This paper outlines the design of an eDelphi study to address the lack of research and evidence base for patient safety interventions in FCV settings. The aim of the study is to seek an

For peer review only - http://bmjopen.bmj.com/site/about/guidelines.xhtml

understanding and gain consensus on the interventions that may be most relevant in FCV settings. Recognising the increasing scope of FCV settings, and their wide-ranging nature and impact on increasing numbers of the global population, this is timely work. It also holds a crucial place within the UHC agenda, whilst access to healthcare for all and improved equity of provision is unequivocally a priority, the development of health systems, particularly within LMICs and further FCV settings needs to be undertaken alongside adequate attention to the safety and quality of care that is provided to ensure that access to healthcare also results in improved health outcomes and not further harm.

It is hoped that the results of this study will be able to be utilised to create a list of the most relevant patient safety interventions, based on the consensus reached among experts with experience of, or working in the field. The outcomes of this study have the potential to increase awareness in this area, and to identify interventions with a higher priority for implementation and further evaluation and research.

Strengths and Limitations

There are both strengths and limitations for this study. The qualitative aspect of the study will enable the collection of detailed information from participants to answer the research question, and in addition may include the collation of relevant information that may not have been anticipated by the researchers [13]. It also offers the opportunity for the research team to explore participants' perceptions and the justifications for their responses in detail [14]. The use of a Delphi method specifically offers flexibility to adapt the research to reflect the unique and challenging topic, offers anonymity to the participants, and the opportunity to probe and develop existing knowledge and areas of controversy collectively to better clarify and distil perspectives [15, 16].

The nature of the eDelphi we intend to employ and the sampling strategy adopted will also enable the contribution of geographically diverse participants from regions around the world, and a good representation of those working in and with lived experience of providing healthcare in these settings. The online nature of the eDelphi is further valuable in the context of the ongoing COVID-19 pandemic, as participation in the consensus building exercise need not be restricted by distancing as part of infection and prevention control. Contributions will remain anonymous and will not be individually identifiable to encourage participants to name barriers and challenges to implementation of interventions.

For peer review only - http://bmjopen.bmj.com/site/about/guidelines.xhtml

There are also a number of limitations to this work (see Table 1). Common critiques of the Delphi method tend to focus on the challenge of defining consensus, which can vary substantially from one Delphi research project to another, and the attrition rate of participants, which typically increases in each round of the study [17]. In conducting research in FCV settings, we would expect attrition rates to be particularly high due to the nature of work and conflicting priorities among participants. We will seek to mitigate this challenge by addressing the time commitment required from participants, including reducing the number of rounds and length of surveys where possible. Similarly, in the first instance we will seek a larger sample size to account for attrition.

The Delphi method has also been criticised for the role of response anonymity and the subsequent lack of participant accountability and ownership of ideas, which is further exacerbated in the eDelphi technique as physical anonymity is an additional characteristic [15,16,17]. Further, in seeking consensus on topics in FCV settings, participants unable to be in the same room to discuss their experiences and how these have shaped their responses and debate differences of opinion may disengage with the process. We will seek to be mindful of this challenge and address any participant concerns through the course of the research. The nature of the eDelphi, specifically the use of online platform Qualtrics to administer the survey questionnaire instead of a traditional face-to-face approach, will require a stable internet source and internet access. Given the internet access and connectivity challenges in some of the most challenging FCV settings, some potential participants in the survey may be excluded from participating in the research. However, in Table 1 we outline several mitigation strategies noted by Donohoe et al. (2012) that may be relevant in the FCV content [16].

For peer review only - http://bmjopen.bmj.com/site/about/guidelines.xhtml

**Select Delphi study limitations**	**Reflections on limitations in the FCV research content**	**Mitigation strategies in the FCV research context**
Challenge in defining consensus	No unique challenges in defining consensus in FCV research.	A clear plan to define consensus, based on best practice and in line with published research, agreed by the research team could be beneficial at the protocol development stage.
High attrition rates	Attrition rates in FCV research are likely to be higher than in other areas. Individuals involved in FCV research working in FCV settings will likely have a number of competing priorities due to a lack of healthcare resources in these settings.	Attrition rates should be considered in protocol development, with steps taken to reduce the number of rounds and length of questionnaires to capture only the information required for the study.
Anonymity and accountability	Anonymity may play a more complex role in FCV research as the information provided and discussed by participants is likely to be emotive and may involve the sharing of negative experiences in justifying judgements.	The research team should be mindful of the negative aspects of anonymity in Delphi studies and work with participants to ensure that the sharing of personal experiences by participants and analysis of these are undertaken in a respectful manner with particular attention to the

For peer review only - http://bmjopen.bmj.com/site/about/guidelines.xhtml

		emotive nature of the topic and participant well-being.
Internet access	Internet access challenges for some participants in FCV settings is likely to be a challenge. In FCVs connectivity may be poor or non-existent, excluding individuals in these settings from participating in the study.	Depending on the nature of the research project and funding available there are several mitigation strategies that could be employed. For example, Donohoe et al. (2012) suggest making paper surveys available to participants with no internet access / limited access or offering financial assistance to facilitate access where appropriate [16].

For peer review only - http://bmjopen.bmj.com/site/about/guidelines.xhtml

Loss of research control	In FCV settings there are additional risks in losing research control, specifically participants missing deadlines, due to a range of possible distractions and delays to participation. Additionally, the security of data related to individual participation may be more challenging in the FCV environment.	The research team could develop a risk register outlining risks to maintaining control. Additionally, Donohoe et al. (2012) note the value of identifying possible distractions/delays to participation [16]. In the FCV setting, FCV-based disruptions (e.g. political challenges, weather challenges) should also be considered.<br>Similarly, Donohoe et al. (2012) recommend ensuring secure survey access through unique passwords and hyperlinks [16]. In the FCV setting this may be increasingly important as systems may have lower cybersecurity protection in place and healthcare organisations are frequently the target of attacks (both physical and cyber) [18,19].

Table 1. Reflections on limitations and their mitigation in the FCV research context

Finally, the eDelphi has been criticised for the inherent loss of research control as the research is moved online, giving the research team less oversight on appropriate participation, including ensuring "expert" participation, and adherence to protocols [16]. As highlighted in Table 1, the

For peer review only - http://bmjopen.bmj.com/site/about/guidelines.xhtml

development of a risk register may offer the research team a lens by which to assess the major threats to research control in the FCV setting, including but not limited to, FCV-based disruptions and security challenges.

Conclusion / Dissemination of results

It is hoped that the results of this work can provide guidance to front line organisations, providers, and policy makers. We will seek to present the findings of this work to the World Health Organization in the hope it can help to inform future work streams. The work will further inform the WHO National Quality Policy and Strategy (NQPS) Initiative [20]. The results will provide direction for future bodies of work on patient safety interventions and bring increased awareness and funding to this area. It may also influence policy to include patient safety as a crucial aspect of health systems strengthening and progress towards UHC. A research paper based on the study results will also be submitted to peer-reviewed journals to be considered for publication.

Author contributions

ALN, AS and NO contributed to the conception and design of this work and wrote the manuscript with input from all authors. All authors approved the version submitted for publication.

Funding statement

This work was supported by the National Institute for Health Research (NIHR) Imperial Patient Safety Translation Research Centre. Infrastructure support was provided by the NIHR Imperial Biomedical Research Centre (BRC).

Competing interests

None declared.

For peer review only - http://bmjopen.bmj.com/site/about/guidelines.xhtml

Data sharing statement

Not applicable

Patient consent

Not required.

Disclaimer

The views of the authors do not necessarily reflect those of the NHS, NIHR or the Department of Health.

Provenance and peer review

Not commissioned; externally peer reviewed.

Acknowledgements

This work has not been previously published.

For peer review only

References

1. National Academies of Sciences Engineering and Medicine (NASEM). Crossing the Global Quality Chasm: Improving Health Care Worldwide. 2018. Washington DC. Available from: doi:10.17226/25152
2. Rice H E, Lou-Meda R, Saxton AT, Johnston BE, Ramirez CC, Mendez S, et al. Building a safety culture in global health: lessons from Guatemala. BMJ Global Health. 2018;3. Available from: doi:10.1136/bmjgh-2017-9996390
3. Syed S B, Leatherman S, Neilson M, Griekspoor A, Horemans D, Letaief M, et al. Improving quality of care in fragile, conflict-affected and vulnerable settings. Bull World Health Organ. 2020 Jan 1;98(1):2-2A. Available from: doi: 10.2461/BLT.19.246280
4. Quality of care in fragile, conflict-affected and vulnerable settings: taking action. Geneva: World Health Organization; 2020. Licence. CC BY-NC-SA 3.0 IGO.
5. Leatherman S, Tawfik L, Jaff D, Jaworski G, Neilson M, Letaief M et al. Quality healthcare in extreme adversity: Developing a framework for action. International Journal for Quality in Health care. 2020; 32 (2), 149-155. Available from: doi:10.1093/intqhc.mzz131
6. WHO | WHO Surgical Safety Checklist [Internet]. WHO. World Health Organization. Available from: http://www.who.int/patientsafety/topics/safe-surgery/checklist/en/ [cited 2021 Apr 21].
7. Haynes A B, Weiser T G, Berry W R, Lipsitz S R, Breizat A-HS, Dellinger E P, et al. A Surgical Safety Checklist to Reduce Morbidity and Mortality in a Global Population. New England Journal of Medicine. 2009 Jan 29;360(5):491–9. Available from: doi:10.1056/NejMsa0810119
8. Keeney S, Hasson F, McKenna H P. A critical review of the Delphi technique as a research methodology for nursing. Int J Nurs Stud 2001; 38:195–200. Available from:10.1016/S0020-7489(00)000444-4
9. Keeney S, Hasson F, McKenna H. Consulting the oracle: ten lessons from using the Delphi technique in nursing research. J Adv Nurs 2006; 53:205–12. Available from: doi:10.1111/j.1365-2648.2006.03716.x
10. Braun V. & Clarke V. Using thematic analysis in psychology. Qualitative research in psychology 2006; 3:77–101. Available from: doi:10.1191/1478088706qp063oa

For peer review only - http://bmjopen.bmj.com/site/about/guidelines.xhtml

11. Ritchie J, Spencer L. Qualitative data analysis for applied policy research. In Analysing Qualitative Data. London: Routledge; 1994. Available from: doi:10.4135/9781412986274.n12
12. Tong A, Sainsbury P, Craig J. Consolidated criteria for reporting qualitative research (COREQ): a 32-item checklist for interviews and focus groups. Int J Qual Health Care. 2007;19(6):349-357. Available from: doi:10.1093/intqhc/mzm042
13. Pope C, Royen P van, Baker R. Qualitative methods in research on healthcare quality. BMJ Quality & Safety. 2002 Jun 1;11(2):148–52. Available from: doi:10.1136/qhc.11.2.148
14. Fitzpatrick R, Boulton M. Qualitative methods for assessing healthcare. Quality in Health Care. 1994. 3: 107-113. Available from: doi:10.1136/qshc.3.2.107
15. Fink-Hafer D, Dagen T, Doušak M, Novak M, Hafner-Fink M. Delphi Method: Strengths and Weaknesses. Metodološki zvezki. 2019;16 (2): 1-19. Available from: https://www.researchgate.net/publication/337570516_Delphi_Method_Strengths_and_Weaknesses [cited 2021 Apr 21].
16. Donohoe H, Stellefson M, Tennant B. Advantages and Limitations of the e-Delphi Technique, American Journal of Health Education. 2013; (1): 38-46. Available from: doi:10.1080/19325037.2012.10599216
17. Shariff N J. Utilizing the Delphi Survey Approach: A Review. J Nurs Care. 2015; 4 (3). Available from: doi:10.4172/2167-1168.1000246.
18. International Committee of the Red Cross. *What limits does the law of war impose on cyber attacks?* Available from: https://www.icrc.org/en/doc/resources/documents/faq/130628-cyber-warfare-q-and-a-eng.htm [cited 2021 April 21].
19. International Committee of the Red Cross. *Call to governments: Work together to stop cyber attacks on health care.* Available from: https://www.icrc.org/en/document/governments-work-together-stop-cyber-attacks-health-care [cited 2021 April 21].
20. World Health Organization. *National quality policy and strategy.* Available from: https://www.who.int/teams/integrated-health-services/quality-health-services/national-quality-policy-and-strategy [cited 2021 April 21].

For peer review only - http://bmjopen.bmj.com/site/about/guidelines.xhtml

BMJ Open

How to improve patient safety in fragile, conflict-affected and vulnerable settings: a Delphi study protocol

Journal:	*BMJ Open*
Manuscript ID	bmjopen-2021-052960.R1
Article Type:	Protocol
Date Submitted by the Author:	19-Jul-2021
Complete List of Authors:	Shaw, Alexandra ; Imperial College London, Patient Safety Translational Research Centre<br>O'Brien, Niki; Imperial College London, Institute of Global Health Innovation<br>Flott, Kelsey; Imperial College London,<br>Leatherman, Sheila; University of North Carolina at Chapel Hill Gillings School of Global Public Health<br>Durkin, Michael; Imperial College London, Institute for Global Health Innovation; Imperial College London, Centre for Health Policy, Institute of Global Health Innovation<br>Darzi, Ara; Imperial College London, Institute of Global Health Innovation<br>Neves, Ana Luisa; Imperial College London,
**Primary Subject Heading**:	Global health
Secondary Subject Heading:	Research methods, Health policy, Health services research
Keywords:	HEALTH SERVICES ADMINISTRATION & MANAGEMENT, Protocols & guidelines < HEALTH SERVICES ADMINISTRATION & MANAGEMENT, Organisation of health services < HEALTH SERVICES ADMINISTRATION & MANAGEMENT, International health services < HEALTH SERVICES ADMINISTRATION & MANAGEMENT, Quality in health care < HEALTH SERVICES ADMINISTRATION & MANAGEMENT, QUALITATIVE RESEARCH

SCHOLARONE™
Manuscripts

I, the Submitting Author has the right to grant and does grant on behalf of all authors of the Work (as defined in the below author licence), an exclusive licence and/or a non-exclusive licence for contributions from authors who are: i) UK Crown employees; ii) where BMJ has agreed a CC-BY licence shall apply, and/or iii) in accordance with the terms applicable for US Federal Government officers or employees acting as part of their official duties; on a worldwide, perpetual, irrevocable, royalty-free basis to BMJ Publishing Group Ltd ("BMJ") its licensees and where the relevant Journal is co-owned by BMJ to the co-owners of the Journal, to publish the Work in this journal and any other BMJ products and to exploit all rights, as set out in our licence.

The Submitting Author accepts and understands that any supply made under these terms is made by BMJ to the Submitting Author unless you are acting as an employee on behalf of your employer or a postgraduate student of an affiliated institution which is paying any applicable article publishing charge ("APC") for Open Access articles. Where the Submitting Author wishes to make the Work available on an Open Access basis (and intends to pay the relevant APC), the terms of reuse of such Open Access shall be governed by a Creative Commons licence – details of these licences and which Creative Commons licence will apply to this Work are set out in our licence referred to above.

Other than as permitted in any relevant BMJ Author's Self Archiving Policies, I confirm this Work has not been accepted for publication elsewhere, is not being considered for publication elsewhere and does not duplicate material already published. I confirm all authors consent to publication of this Work and authorise the granting of this licence.

For peer review only

How to improve patient safety in fragile, conflict-affected and vulnerable settings: a Delphi study protocol

Alexandra Shaw[1], Niki O'Brien[2], Kelsey Flott[1], Sheila Leatherman[3], Mike Durkin[1], Ara Darzi[1], Ana Luisa Neves[1,4]

[1] Imperial NIHR Patient Safety Translational Research Centre, Institute of Global Health Innovation, Department of Surgery and Cancer, Imperial College London, London, UK

[2] Institute of Global Health Innovation, Imperial College London, London, UK

[3] UNC Gillings School of Global Public Health, University of North Carolina

[4] Department of Community Medicine, Information and Health Decision Sciences (MEDCIDS) / Center for Health Technology and Services Research (CINTESIS), University of Porto, Porto, Portugal

Corresponding author

Niki O'Brien

Institute of Global Health Innovation,

QEQM Wing, St Mary's Campus,

London W2 1NY

n.obrien@imperial.ac.uk

Keywords: Patient Safety, fragile, conflict-affected and vulnerable settings; FCVs; organisation of health services; international health services; quality in healthcare; qualitative research; research methods; protocol; Delphi study.

Word count: 3,198

For peer review only

Abstract

Introduction: There is a high burden of adverse events and poor outcomes in fragile, conflict-affected, and vulnerable (FCV) settings. Thus far, there has been limited research on the improvement of patient safety and implementation of interventions in FCV settings.

Objective: This study intends to generate a consensus on the most relevant and applicable patient safety interventions for these settings from experts on FCV settings, including: front line clinicians and managers/administrators, NGOs, policy makers, and researchers, and gather information on the enabling factors and barriers for their effective implementation.

Methods and Analysis: The study utilises a Delphi research approach, specifically an eDelphi. This protocol outlines research that will be conducted online with expert participants from a range of backgrounds, including those working in a variety of FCV settings. Participants will be established contacts known to the research team or recruited via snowballing sampling. Research will be conducted across three rounds, with each round taking approximately 15 minutes to complete. Consensus will be considered reached based on a defined minimum mean score and either an interquartile range or a percentage of participants who gave the set minimum mean score. Data analysis will be completed on Microsoft Excel and NVivo. The primary outcome of the study will be a list of the most relevant and applicable patient safety interventions for FCV settings.

Ethics and dissemination: The study has received approval from Imperial College London Ethics Committee reference number 20IC665. Anonymous results will be made available to the public, academic organisations, and policymakers.

For peer review only

Strengths and limitations of this study

- A key strength of this study is the utilisation of an eDelphi approach offering flexibility and anonymity to facilitate more open discussion and the ability to conduct research in the COVID-19 era without transmission risk for participants.
- This study has the potential to start to build an evidence base for which interventions could be developed further and evaluation undertaken.
- This study has the potential to draw attention and funding to this area and contribute to policy and real-world interventions to improve the safety of care in FCV settings.
- Limitations of the study include the potential exclusion of those working in the field due to unreliable or lack of internet access.
- We will attempt to mitigate higher attrition rates among participants, due to the nature of the work and conflicting priorities, with careful consideration of the participant list and by having as few rounds and short length of survey as possible.

For peer review only

For peer review only - http://bmjopen.bmj.com/site/about/guidelines.xhtml

Introduction

The safety of patients is a critical element of well-functioning health systems globally and is an essential component of improving health outcomes and achieving universal health coverage (UHC). Whilst the focus of health system strengthening has historically been on UHC, there has been increasing acknowledgement in recent years that without improving safety and a goal to achieve high quality care, healthcare will not provide the desired outcomes. In low- and middle-income countries (LMICs) attention to patient safety is particularly important given the prevalence of patient safety incidents in these settings; accounting for an estimated 134 million adverse events, and 2.5 million deaths annually [1]. While the need to improve patient safety is an increasingly recognised priority within the global health agenda, there is still much to be done to develop and implement appropriate tools to achieve this goal [2].

The term fragile, conflict-affected and vulnerable (FCV) settings describes any setting of crisis, and may include armed conflict, complex emergencies, natural disasters and disruption to public services, and is recognised by the World Health organisation and The World Bank [3][4][5]. The World Health Organization (WHO) publication *Quality of care in fragile, conflict-affected and vulnerable settings* (2020) defines FCV settings as settings: "experiencing humanitarian crises, protracted emergencies, prolonged disruption to critical public services or governance (e.g. due to political or economic challenges, conflict or natural disaster), or armed conflict." [4]. The World Bank publishes an annual list of fragile and conflict-affected situations [5]. Despite their heterogeneous nature, FCV settings share common challenges, including the disruption of routine health service organisation and delivery systems, increased health needs, complex resourcing landscapes, and vulnerability to further public health crises" [4].

Approximately 2 billion people are currently estimated to live in FCV settings with this figure set to rise [6]. Estimates suggest large numbers of preventable deaths take place in FCV settings including; 60% of preventable maternal deaths, 53% of deaths in children under five years, and 45% of neonatal deaths [4]. Patient safety priorities and the facilitators and barriers to implementing patient safety interventions vary between and across individual FCV settings, reflecting the need for a flexible approach to improving safety and quality in service delivery [4].

To reduce the impact of unsafe care on patient outcomes in FCV settings, patient safety interventions could be implemented to reduce incidents and improve the standard of care. Incidents

For peer review only - http://bmjopen.bmj.com/site/about/guidelines.xhtml

of unsafe care range from wrong site surgery to incorrect patient identification and delayed or missed opportunities for intervention resulting in preventable morbidity and mortality [4]. Potential interventions cover a range of areas including, for example, infection prevention and control, health worker training and point of care, however barriers to implementation can be considerable in these settings.

An example of a specific intervention is the WHO Surgical Safety Checklist which, in a study by Haynes et al. (2009), the rate of death before and after the checklist was introduced declined from 1.5% to 0.8% and inpatient complications dropped from 11% to 7%. [7,8]. Notably, the implementation of the surgical checklist requires a context specific approach to maximise patient safety improvements [9], and as such, greater research is required in different FCV settings to understand the facilitators and barriers to successful implementation in different contexts.

Despite the significant impact of patient safety incidents in FCV settings, there is limited evidence about the most applicable patient safety interventions, and how best to support these communities to improve the safety of care. Such a gap in knowledge makes it challenging to assess the quality and safety of existing interventions, measure their accessibility and effectiveness, address any context specific challenges, and develop new interventions [4]. In other healthcare settings it is possible to capture data and record it over time, however in FCV settings this can be challenging due to system, local or organisational leadership instability, lack of technology and infrastructure and clinical priorities due to the demand for care on limited resources and staff. As such, a critical starting point must be to build an evidence base and identify which interventions are considered most useful and relevant in FCV settings and furthermore what is required to develop such interventions to be accessible and effective. It is unlikely that this evidence will initially come from traditional academic sources, particularly with the exacerbation of fragility caused by the COVID-19 pandemic, however there is an opportunity to bring together and build on the knowledge held in the experiences of experts and practitioners working in these contexts.

The aim of the study is to identify the most relevant patient safety interventions for these settings and also gather information on the enabling factors and barriers for their implementation. The Delphi study design used by the research team sought to gain consensus on the question 'What is needed to improve patient safety in FCV settings?' from clinicians working on the ground,

For peer review only - http://bmjopen.bmj.com/site/about/guidelines.xhtml

managers/administrators, policy makers, researchers and non-governmental organizations (NGOs).

Methods and analysis

Study design

The study will use a Delphi methodology to seek consensus on a question among a group of experts. Where there is a lack of research evidence and a desire to reach consensus, a commonly used formal consensus method is the Delphi technique, which uses rounds of questionnaires to collect data and achieve group consensus [10]. As the research will be conducted remotely online, through this paper we utilise both the term "Delphi", to describe the established research technique, and the term "eDelphi" to describe our proposed research in the FCV setting.

The eDelphi study will seek to gain consensus on the question 'Which interventions are needed to improve patient safety in FCV settings?'. For the purposes of the study "interventions" will be defined in the survey as "the act of interfering with the outcome or course especially of a condition or process (as to prevent harm or improve functioning)" [11]. Qualtrics will be used to develop each round of the online questionnaire survey. The survey will be conducted across three rounds and each round of the survey will take approximately 15 minutes or less to complete. A link to each survey will be distributed via email to all participants followed by two reminder emails, at one-week intervals, per survey round.

In round 1, participant demographics will also be collected, including: gender, year of birth, country of residence, and current professional role. In Rounds 1-2, experts will be asked to rank the thematic areas on a Likert scale (1-4, without 0). Participants will also be asked to determine the accuracy of the thematic areas of patient safety interventions outlined on a four-point Likert scale ('strongly agree', 'agree', 'disagree', 'strongly disagree'). The four-point Likert scale has been utilised in previous Delphi studies, producing stable findings [12]. For each statement, participants will be given the option to select 'don't know' as an alternative response. A free-text response will also be made available to participants after each statement, facilitating them to elaborate on their thoughts or explain their responses. In Round 3, experts will be presented with

For peer review only - http://bmjopen.bmj.com/site/about/guidelines.xhtml

the individual and group results from Round 2, to enable them to reconsider their responses in this round. With this information, participants will be asked to order each of the interventions that received consensus in Rounds 1 and 2 based on which are likely to be most impactful. Responses will be subsequently analysed and ultimately mapped in thematic areas ranked by importance.

Survey development

Statements for the survey were developed from the study team's expertise and a review of the literature . As part of the preliminary work, we will undertake a rapid literature review and ask the experts who will participate in the subsequent eDelphi to answer an exploratory question to list as many interventions as they want or a set number of responses to the question: 'Which interventions are needed to improve patient safety in FCV settings?'. The questionnaire will be hosted on Qualtrics, with a link to the survey distributed via email to all participants followed by two reminder emails, at one-week intervals. The data will be analysed by two independent qualitative researchers using thematic analysis (NVivo Software). To cross-check data analysis and ensure data quality, consistency in approach, and transparency of analytical decision making, 50% of the data will be read and coded independently by a second experienced qualitative researcher [13]. Discrepancies in interpretation will be reviewed and resolved by a third qualitative researcher. A number of statements representing each theme will then be developed.

Expert panel recruitment

Delphi studies employ a panel of expert members, as opposed to a random representative sample. In this study, 'expert' will be defined as a professional with experience working in health delivery and/or patient safety in FCV settings. For the purposes of recruitment in the study, "FCV settings" will be considered as per the definition of the WHO (i.e. settings experiencing humanitarian crises, protracted emergencies, prolonged disruption to critical public services or governance (e.g., due to political or economic challenges, conflict or natural disaster), or armed conflict."[4] These settings can be entire countries (e.g., Syria) where war has affected the national health system, or where only some geographic areas of countries have been affected (e.g., the aftermath of a natural disaster, an influx of refugees to a particular area etc.) This definition enables the recruitment of experts across high, middle- and low-income health systems to get a broad range of opinions (see Figure 1). We will recruit experts on the countries listed in the World Bank FY22 List of Fragile

For peer review only - http://bmjopen.bmj.com/site/about/guidelines.xhtml

and Conflict-affected Situations, as well as individuals working in these settings [5]. We will also recruit experts who currently work, or have previously worked, in settings at the intersection outlined in Figure 1. Participants will be included if aged 18 years old or older and able to understand English.

[Figure 1 around here]

Figure 1: Conceptual framework for expert panel recruitment

Purposive sampling will be used to ensure representativeness from a variety of expert roles (clinicians, managers/administrators, policymakers, researchers, and NGOs) at the organisational and governmental level. The majority of participants will be established contacts known to the research team via the Institute of Global Health Innovation's (IGHI) Leading Health Systems Network (LHSN) and the NIHR Imperial Patient Safety Translational Research Centre (PSTRC). Other relevant participants will be identified through the team's contacts at the World Health Organization (WHO) or using snowballing sampling. The use of snowball sampling is needed due to this being a diverse field, with no centralised community of practice across FCV settings. The team members have previous research and an extensive network to access contacts in these areas. Attention will be paid to ensure the range of participants is geographically diverse and reflects the wide range of FCV settings, including settings of current or recent armed conflict, complex emergencies, natural disasters, and disruption to public services.

There is no agreement in the literature on the optimal number of participants to include in a Delphi survey [14]. Therefore, we will include all 'experts' who express an initial interest to participate. An initial invitation email will be sent to possible participants identified by the research team. Participants will be made aware that their involvement is voluntary and if they do not take part in the study it will not affect their relationship with the study team. Each participant will be given a Participant Information Sheet and provided with the opportunity to address any queries they may have with a researcher.

Data analysis

Descriptive statistics will be used to describe participants' characteristics, as well as their responses to the survey questions, including means, medians, standard deviation (SD) and

For peer review only - http://bmjopen.bmj.com/site/about/guidelines.xhtml

interquartile range (IQR). Consensus will be defined as > 70% of participants agreeing/strongly agreeing or disagreeing/strongly disagreeing with a statement. This level of agreement has been previously considered appropriate in similar Delphi studies [15][16]. "Don't know" answers will be excluded from the analysis.

Patient and public involvement

Patient partners will be included in the interpretation of our results, in the co-development of a dissemination strategy, and in summarising the research findings into lay summaries and reports, in order to raise awareness and stimulate public participation on this topic. The research team at the Imperial NIHR PSTRC has a dedicated PPIE lead and a Research Partners Group.

Ethics and dissemination

This project has received ethics approval from the Imperial College London Ethics Committee, reference number 20IC665. Consent has been incorporated into the survey alongside the participant information provided.

The results of this study will be published in a peer-reviewed journal and the intention is that this will begin to build an evidence base for further research and provide background for future grant applications. It would also be hoped that the findings of this study will be disseminated via presentation to international audiences through conferences. It is intended that this study will inform future translational research and provide a starting point to develop in-country partnership to increase capacity building for research and patient safety intervention implementation in these settings.

Discussion

This paper outlines the design of an eDelphi study to address the lack of research and evidence base for patient safety interventions in FCV settings. The aim of the study is to seek an understanding and gain consensus on the interventions that may be most relevant in FCV settings. Recognising the increasing scope of FCV settings, and their wide-ranging nature and impact on increasing numbers of the global population, this is timely work. It also holds a crucial place within the UHC agenda, whilst access to healthcare for all and improved equity of provision is

For peer review only - http://bmjopen.bmj.com/site/about/guidelines.xhtml

unequivocally a priority, the development of health systems, particularly within LMICs and further FCV settings needs to be undertaken alongside adequate attention to the safety and quality of care that is provided, to ensure that access to healthcare also results in improved health outcomes and not further harm.

It is hoped that the results of this study will be able to be utilised to create a list of the most relevant patient safety interventions, based on the consensus reached among experts with experience of, or working in the field. The outcomes of this study have the potential to increase awareness in this area, and to identify interventions with a higher priority for implementation and further evaluation and research.

Strengths and Limitations

There are both strengths and limitations for this study. The qualitative aspect of the study will enable the collection of detailed information from participants to answer the research question, and in addition may include the collation of relevant information that may not have been anticipated by the researchers [17]. It also offers the opportunity for the research team to explore participants' perceptions and the justifications for their responses in detail [18]. The use of a Delphi method specifically offers flexibility to adapt the research to reflect the unique and challenging topic, offers anonymity to the participants, and the opportunity to probe and develop existing knowledge and areas of controversy collectively to better clarify and distil perspectives [19, 20].

The nature of the eDelphi we intend to employ, and the sampling strategy adopted, will also enable the contribution of geographically diverse participants from regions around the world, and a good representation of those working in and with lived experience of providing healthcare in these settings. The online nature of the eDelphi is further valuable in the context of the ongoing COVID-19 pandemic, as participation in the consensus building exercise need not be restricted by distancing as part of infection and prevention control. Contributions will remain anonymous and will not be individually identifiable to encourage participants to name barriers and challenges to implementation of interventions.

There are also a number of limitations to this work (see Table 1). Common critiques of the Delphi method tend to focus on the challenge of defining consensus, which can vary substantially from one Delphi research project to another, and the attrition rate of participants, which typically

For peer review only - http://bmjopen.bmj.com/site/about/guidelines.xhtml

increases in each round of the study [21]. In conducting research in FCV settings, we would expect attrition rates to be particularly high due to the nature of work and conflicting priorities among participants. We will seek to mitigate this challenge by addressing the time commitment required from participants, including reducing the number of rounds and length of surveys where possible. Similarly, in the first instance we will seek a larger sample size to account for attrition.

The Delphi method has also been criticised for the role of response anonymity and the subsequent lack of participant accountability and ownership of ideas, which is further exacerbated in the eDelphi technique as physical anonymity is an additional characteristic [19,20,21]. Further, in seeking consensus on topics in FCV settings, participants unable to be in the same room to discuss their experiences and how these have shaped their responses and debate differences of opinion may disengage with the process. We will seek to be mindful of this challenge and address any participant concerns through the course of the research. The nature of the eDelphi, specifically the use of online platform Qualtrics to administer the survey questionnaire instead of a traditional face-to-face approach, will require a stable internet source and internet access. Given the internet access and connectivity challenges in some of the most challenging FCV settings, some potential participants in the survey may be excluded from participating in the research. However, in Table 1 we outline several mitigation strategies noted by Donohoe et al. (2012) that may be relevant in the FCV content [20].

**Select Delphi study limitations**	**Reflections on limitations in the FCV research content**	**Mitigation strategies in the FCV research context**
Challenge in defining consensus	No unique challenges in defining consensus in FCV research.	A clear plan to define consensus, based on best practice and in line with published research, agreed by the research team could be beneficial at the protocol development stage.

For peer review only - http://bmjopen.bmj.com/site/about/guidelines.xhtml

High attrition rates	Attrition rates in FCV research are likely to be higher than in other areas. Individuals involved in FCV research working in FCV settings will likely have several competing priorities due to a lack of healthcare resources in these settings.	Attrition rates should be considered in protocol development, with steps taken to reduce the number of rounds and length of questionnaires to capture only the information required for the study.
Anonymity and accountability	Anonymity may play a more complex role in FCV research as the information provided and discussed by participants is likely to be emotive and may involve the sharing of negative experiences in justifying judgements.	The research team should be mindful of the negative aspects of anonymity in Delphi studies and work with participants to ensure that the sharing of personal experiences by participants and analysis of these are undertaken in a respectful manner with particular attention to the emotive nature of the topic and participant well-being.
Internet access	Internet access challenges for some participants in FCV settings is likely to be a challenge. In FCVs connectivity may be poor or non-existent, excluding individuals in these settings from participating in the study.	Depending on the nature of the research project and funding available there are several mitigation strategies that could be employed. For example, Donohoe et al. (2012) suggest making paper surveys available to participants with no internet access / limited access or

For peer review only - http://bmjopen.bmj.com/site/about/guidelines.xhtml

		offering financial assistance to facilitate access where appropriate [20].

For peer review only

For peer review only - http://bmjopen.bmj.com/site/about/guidelines.xhtml

Loss of research control	In FCV settings there are additional risks in losing research control, specifically participants missing deadlines, due to a range of possible distractions and delays to participation. Additionally, the security of data related to individual participation may be more challenging in the FCV environment.	The research team could develop a risk register outlining risks to maintaining control. Additionally, Donohoe et al. (2012) note the value of identifying possible distractions/delays to participation [20]. In the FCV setting, FCV-based disruptions (e.g., political challenges, weather challenges) should also be considered.<br>Similarly, Donohoe et al. (2012) recommend ensuring secure survey access through unique passwords and hyperlinks [20]. In the FCV setting this may be increasingly important as systems may have lower cybersecurity protection in place and healthcare organisations are frequently the target of attacks (both physical and cyber) [22,23].

Table 1. Reflections on limitations and their mitigation in the FCV research context

Finally, the eDelphi has been criticised for the inherent loss of research control as the research is moved online, giving the research team less oversight on appropriate participation, including ensuring “expert” participation, and adherence to protocols [20]. As highlighted in Table 1, the

For peer review only - http://bmjopen.bmj.com/site/about/guidelines.xhtml

development of a risk register may offer the research team a lens by which to assess the major threats to research control in the FCV setting, including but not limited to, FCV-based disruptions and security challenges.

Author contributions

ALN, AS and NO conceptualised the manuscript. ALN planned the development of the work and ALN, AS and NO led in the writing of the original draft manuscript, with input KF, SL, MD, and AD. All authors (AS, NO, KF, SL, MD, AD, ALN) equally contributed to the writing, reviewing, and editing to develop the final draft. All authors approved the version submitted for publication.

Funding statement

ALN supported by the National Institute for Health Research (NIHR) Imperial Patient Safety Translation Research Centre (Award reference is PSTRC_2016_004). Infrastructure support was provided by the NIHR Imperial Biomedical Research Centre (BRC).

Competing interests

None declared.

Data sharing statement

Not applicable

Patient consent

Not required.

Disclaimer

For peer review only

The views of the authors do not necessarily reflect those of the NHS, NIHR or the Department of Health.

Provenance and peer review

Not commissioned; externally peer reviewed.

Acknowledgements

This work has not been previously published.

For peer review only

References

1. National Academies of Sciences Engineering and Medicine (NASEM). Crossing the Global Quality Chasm: Improving Health Care Worldwide. 2018. Washington DC. Available from: doi:10.17226/25152
2. Rice H E, Lou-Meda R, Saxton AT, Johnston BE, Ramirez CC, Mendez S, et al. Building a safety culture in global health: lessons from Guatemala. BMJ Global Health. 2018;3.
3. Syed S B, Leatherman S, Neilson M, Griekspoor A, Horemans D, Letaief M, et al. Improving quality of care in fragile, conflict-affected and vulnerable settings. Bull World Health Organ. 2020 Jan 1;98(1):2-2A.
4. Quality of care in fragile, conflict-affected and vulnerable settings: taking action. Geneva: World Health Organization; 2020.
5. World Bank. Classification of Fragile and Conflict-Affected Situations [Internet]. World Bank; 2021. Available from: https://www.worldbank.org/en/topic/fragilityconflictviolence/brief/harmonized-list-of-fragile-situations [cited 15 Jul 21].
6. Leatherman S, Tawfik L, Jaff D, Jaworski G, Neilson M, Letaief M et al. Quality healthcare in extreme adversity: Developing a framework for action. International Journal for Quality in Health care. 2020; 32 (2), 149-155.
7. WHO | WHO Surgical Safety Checklist [Internet]. WHO. World Health Organization. Available from: http://www.who.int/patientsafety/topics/safe-surgery/checklist/en/ [cited 2021 Apr 21].
8. Haynes A B, Weiser T G, Berry W R, Lipsitz S R, Breizat A-HS, Dellinger E P, et al. A Surgical Safety Checklist to Reduce Morbidity and Mortality in a Global Population. New England Journal of Medicine. 2009 Jan 29;360(5):491–9.
9. Weiser TG, Haynes AB. Ten years of the Surgical Safety Checklist. Br J Surg. 2018 Jul;105(8):927-929.
10. Keeney S, Hasson F, McKenna H P. A critical review of the Delphi technique as a research methodology for nursing. Int J Nurs Stud 2001; 38:195–200.
11. Merriam-Webster. "Intervention." Merriam-Webster.com [Internet]. Merriam-Webster, 2021. Web. 15 Jul 2021. Available from: https://www.merriam-

For peer review only - http://bmjopen.bmj.com/site/about/guidelines.xhtml

webster.com/dictionary/intervention [cited 15 Jul 21].

12. Akins RB, Tolson H, Cole BR. Stability of response characteristics of a Delphi panel: application of bootstrap data expansion. BMC Med Res Methodol. 2005;5:37.
13. Braun V. & Clarke V. Using thematic analysis in psychology. Qualitative research in psychology 2006; 3:77–101.
14. Keeney S, Hasson F, McKenna H. Consulting the oracle: ten lessons from using the Delphi technique in nursing research. J Adv Nurs 2006; 53:205–12.
15. Slade SC, Dionne CE, Underwood M, Buchbinder R. Standardised method for reporting exercise programmes: protocol for a modified Delphi study. BMJ Open. 2014;4:e006682.
16. Diamond IR, Grant RC, Feldman BM, Pencharz PB, Ling SC, Moore AM, et al. Defining consensus: a systematic review recommends methodologic criteria for reporting of Delphi studies. J Clin Epidemiol. 2014 Apr;67(4):401-9.
17. Pope C, Royen P van, Baker R. Qualitative methods in research on healthcare quality. BMJ Quality & Safety. 2002 Jun 1;11(2):148–52.
18. Fitzpatrick R, Boulton M. Qualitative methods for assessing healthcare. Quality in Health Care. 1994. 3: 107-113.
19. Fink-Hafer D, Dagen T, Doušak M, Novak M, Hafner-Fink M. Delphi Method: Strengths and Weaknesses. Metodološki zvezki. 2019;16 (2): 1-19. Available from: https://www.researchgate.net/publication/337570516_Delphi_Method_Strengths_and_Weaknesses [cited 2021 Apr 21].
20. Donohoe H, Stellefson M, Tennant B. Advantages and Limitations of the e-Delphi Technique, American Journal of Health Education. 2013; (1): 38-46.
21. Shariff N J. Utilizing the Delphi Survey Approach: A Review. J Nurs Care. 2015; 4 (3).
22. International Committee of the Red Cross. What limits does the law of war impose on cyber attacks? Available from: https://www.icrc.org/en/doc/resources/documents/faq/130628-cyber-warfare-q-and-a-eng.htm [cited 2021 April 21].
23. International Committee of the Red Cross. Call to governments: Work together to stop cyber attacks on health care. Available from: https://www.icrc.org/en/document/governments-work-together-stop-cyber-attacks-health-care [cited 2021 April 21].

For peer review only - http://bmjopen.bmj.com/site/about/guidelines.xhtml

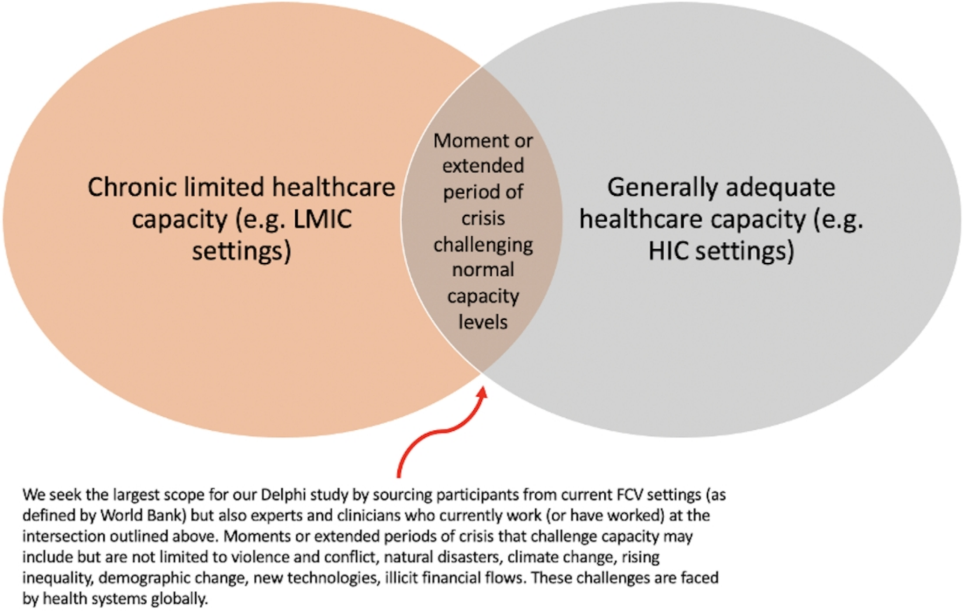

Figure 1: Conceptual framework for expert panel recruitment

165x106mm (300 x 300 DPI)

BMJ Open

How to improve patient safety in fragile, conflict-affected, and vulnerable settings: a Delphi study protocol

Journal:	*BMJ Open*
Manuscript ID	bmjopen-2021-052960.R2
Article Type:	Protocol
Date Submitted by the Author:	22-Sep-2021
Complete List of Authors:	Shaw, Alexandra ; Imperial College London, Patient Safety Translational Research Centre<br>O'Brien, Niki; Imperial College London, Institute of Global Health Innovation<br>Flott, Kelsey; Imperial College London,<br>Leatherman, Sheila; University of North Carolina at Chapel Hill Gillings School of Global Public Health<br>Durkin, Michael; Imperial College London, Institute for Global Health Innovation; Imperial College London, Centre for Health Policy, Institute of Global Health Innovation<br>Darzi, Ara; Imperial College London, Institute of Global Health Innovation<br>Neves, Ana Luisa; Imperial College London,
**Primary Subject Heading**:	Global health
Secondary Subject Heading:	Research methods, Health policy, Health services research
Keywords:	HEALTH SERVICES ADMINISTRATION & MANAGEMENT, Protocols & guidelines < HEALTH SERVICES ADMINISTRATION & MANAGEMENT, Organisation of health services < HEALTH SERVICES ADMINISTRATION & MANAGEMENT, International health services < HEALTH SERVICES ADMINISTRATION & MANAGEMENT, Quality in health care < HEALTH SERVICES ADMINISTRATION & MANAGEMENT, QUALITATIVE RESEARCH

SCHOLARONE™
Manuscripts

I, the Submitting Author has the right to grant and does grant on behalf of all authors of the Work (as defined in the below author licence), an exclusive licence and/or a non-exclusive licence for contributions from authors who are: i) UK Crown employees; ii) where BMJ has agreed a CC-BY licence shall apply, and/or iii) in accordance with the terms applicable for US Federal Government officers or employees acting as part of their official duties; on a worldwide, perpetual, irrevocable, royalty-free basis to BMJ Publishing Group Ltd ("BMJ") its licensees and where the relevant Journal is co-owned by BMJ to the co-owners of the Journal, to publish the Work in this journal and any other BMJ products and to exploit all rights, as set out in our licence.

The Submitting Author accepts and understands that any supply made under these terms is made by BMJ to the Submitting Author unless you are acting as an employee on behalf of your employer or a postgraduate student of an affiliated institution which is paying any applicable article publishing charge ("APC") for Open Access articles. Where the Submitting Author wishes to make the Work available on an Open Access basis (and intends to pay the relevant APC), the terms of reuse of such Open Access shall be governed by a Creative Commons licence – details of these licences and which Creative Commons licence will apply to this Work are set out in our licence referred to above.

Other than as permitted in any relevant BMJ Author's Self Archiving Policies, I confirm this Work has not been accepted for publication elsewhere, is not being considered for publication elsewhere and does not duplicate material already published. I confirm all authors consent to publication of this Work and authorise the granting of this licence.

For peer review only

How to improve patient safety in fragile, conflict-affected, and vulnerable settings: a Delphi study protocol

Alexandra Shaw[1], Niki O'Brien[2], Kelsey Flott[1], Sheila Leatherman[3], Michael Durkin[1], Ara Darzi[1], Ana Luisa Neves[1,4]

[1] Imperial NIHR Patient Safety Translational Research Centre, Institute of Global Health Innovation, Department of Surgery and Cancer, Imperial College London, London, UK

[2] Institute of Global Health Innovation, Imperial College London, London, UK

[3] UNC Gillings School of Global Public Health, University of North Carolina

[4] Department of Community Medicine, Information and Health Decision Sciences (MEDCIDS) / Center for Health Technology and Services Research (CINTESIS), University of Porto, Porto, Portugal

Corresponding author

Niki O'Brien

Institute of Global Health Innovation,

QEQM Wing, St Mary's Campus,

London W2 1NY

n.obrien@imperial.ac.uk

Keywords: Patient Safety, fragile, conflict-affected, and vulnerable settings; FCVs; organisation of health services; international health services; quality in healthcare; qualitative research; research methods; protocol; Delphi study.

Word count: 2,943

For peer review only - http://bmjopen.bmj.com/site/about/guidelines.xhtml

Abstract

Introduction: There is a high burden of adverse events and poor outcomes in fragile, conflict-affected, and vulnerable (FCV) settings. To improve outcomes, there is a need to better identify which interventions can improve patient safety in these settings, as well as to develop strategies to optimise their implementation.

Objective: This study intends to generate a consensus on the most relevant patient safety interventions from experts with experience on FCV settings, including frontline clinicians and managers/administrators, NGOs, policy makers, and researchers.

Methods and Analysis: The study utilises an online Delphi research approach (eDelphi). Participants will include experts from a range of backgrounds, including those working in a variety of FCV settings. Participants will be established contacts known to the research team or recruited via snowballing sampling, and will be asked to identify and rank the importance of a variety of patient safety interventions. Consensus will be defined as > 70% of participants agreeing/strongly agreeing or disagreeing/strongly disagreeing with a statement. Data analysis will be completed in Microsoft Excel and NVivo. The primary outcome of the study will be a list of the most relevant and applicable patient safety interventions for FCV settings.

Ethics and dissemination: The study has received approval from Imperial College London Ethics Committee reference number 20IC665. Anonymous results will be made available to the public, academic organisations, and policymakers.

For peer review only - http://bmjopen.bmj.com/site/about/guidelines.xhtml

Strengths and limitations of this study

- A key strength of this study is the utilisation of an eDelphi approach offering participation of geographically spread participants, and flexibility and anonymity to facilitate open discussion.

- This study has the potential to start building an evidence base and pave the way for the development and evaluation of safety interventions in these settings.

- This study has the potential to draw attention and funding to this area and contribute to policy and real-world interventions to improve the safety of care in FCV settings.

- Limitations of the study include the potential exclusion of those working in the field due to unreliable or lack of internet access.

- We will attempt to mitigate higher attrition rates among participants, due to the nature of the work and conflicting priorities, with careful consideration of the participant list and by having as few rounds as possible, and a short survey lengths.

For peer review only

For peer review only - http://bmjopen.bmj.com/site/about/guidelines.xhtml

Introduction

The safety of patients is a critical element of well-functioning health systems globally - and an essential component to improve health outcomes and sustainably achieve universal health coverage (UHC). Whilst the focus of health system strengthening has historically been on UHC, there has been a growing recognition that the implementation of interventions to improve quality and safety of care is equally critical to achieve better health outcomes. In low- and middle- income countries (LMICs), attention to patient safety is particularly important. In these settings, the prevalence of patient safety incidents is particularly high: every year, it is estimated that 134 million adverse events and 2.5 million deaths occur globally[1]. While the need to improve patient safety is an increasingly recognised priority, there is still much to be done to develop and implement appropriate interventions to achieve this goal [2].

The term fragile, conflict-affected and vulnerable (FCV) settings describes any setting of crisis, and may include armed conflict, complex emergencies, natural disasters and disruption to public services, and is recognised by the World Health Organization and The World Bank [3][4][5]. The World Health Organization (WHO) publication *Quality of care in fragile, conflict-affected and vulnerable settings* (2020) defines FCV settings as settings "experiencing humanitarian crises, protracted emergencies, prolonged disruption to critical public services or governance (e.g. due to political or economic challenges, conflict or natural disaster), or armed conflict."[4] Approximately 2 billion people are currently estimated to live in FCV settings with this figure set to rise [6]. Despite their heterogeneous nature, FCV settings share common challenges, including the "disruption of routine health service organisation and delivery systems, increased health needs, complex resourcing landscapes, and vulnerability to further public health crises" [4]. These factors can pose, both individually and synergically, important threats to the safety of patients. Estimates suggest large numbers of preventable deaths take place in FCV settings, including 60% of preventable maternal deaths, 53% of deaths in children under five years, and 45% of neonatal deaths [4]. Patient safety priorities and the facilitators and barriers to implementing patient safety interventions vary between and across individual FCV settings, reflecting the need for a flexible approach to improving safety and quality in service delivery [4].

To reduce the impact of unsafe care on patient outcomes in FCV settings, patient safety interventions could be implemented to reduce incidents and improve the standard of care. Potential

For peer review only - http://bmjopen.bmj.com/site/about/guidelines.xhtml

interventions cover a range of areas including, for example, infection prevention and control, health worker training and point of care. Incidents of unsafe care range from wrong site surgery to incorrect patient identification and delayed or missed opportunities for intervention resulting in preventable morbidity and mortality [4].

An example of a specific intervention is the WHO Surgical Safety Checklist. In a study by Haynes et al. (2009), the rate of death before and after the checklist was introduced declined from 1.5% to 0.8% and inpatient complications dropped from 11% to 7%. [7,8]. Notably, the implementation of the surgical checklist requires a context specific approach to maximise patient safety improvements [9].

Despite the significant impact of patient safety incidents in FCV settings, there is limited evidence about the most applicable patient safety interventions, and how best to support these communities to improve the safety of care. Such a gap in knowledge makes it challenging to assess the quality and safety of existing interventions, measure their accessibility and effectiveness, address any context specific challenges, and develop new interventions [4]. In other healthcare settings it is possible to capture data and record it over time, however in FCV settings this can be challenging due to system, local or organisational leadership instability, lack of technology and infrastructure and clinical priorities due to the demand for care on limited resources and staff. As such, a critical starting point must be to build an evidence base and identify which interventions are considered most useful and relevant in FCV settings - and to understand what is required to implement interventions that are both accessible and effective. It is unlikely that this evidence will initially come from traditional academic sources, and there is an opportunity to bring together and capitalise on the knowledge and experiences of experts working in these contexts and experts working on this topic area.

The aim of the study is to identify the most relevant patient safety interventions for these settings. The Delphi study design used by the research team will aim to answer the question ‘Which interventions are needed to improve patient safety in FCV settings?”, leveraging on the perspectives of frontline clinicians, managers/administrators, policy makers, researchers, and non-governmental organisations (NGOs).

For peer review only - http://bmjopen.bmj.com/site/about/guidelines.xhtml

Methods and analysis

Study design

The study will use a Delphi methodology to seek consensus among a group of experts. Where there is a lack of research evidence and a desire to reach consensus, a commonly used formal consensus method is the Delphi technique, which uses rounds of questionnaires to collect data and achieve group consensus [10]. As the research will be conducted remotely online, through this paper we utilise both the term "Delphi", to describe the established research technique, and the term "eDelphi" to describe our proposed research.

The eDelphi study will seek to gain consensus on the question 'Which interventions are needed to improve patient safety in FCV settings?'. For the purposes of the study "interventions" will be defined in the survey as "the act of interfering with the outcome or course especially of a condition or process (as to prevent harm or improve functioning)" [11].

Survey development

Statements for the eDelphi (i.e., brief descriptions of the interventions) will be developed from the study team's expertise, a rapid review of the literature, and feedback from the eDelphi participants prior to the start of the Delphi process.

Rapid reviews have been recognised by the World Health Organisation as a useful approach to provide actionable, relevant, and cost-effective evidence [12]. In rapid reviews, the steps of the systematic review are streamlined to produce evidence in a short time frame [12]. In a range of circumstances, including health policy research, there is value in accelerating knowledge synthesis for pressing policy and systems decisions. The keywords used will be made as broad as possible to capture as many publications as possible on the topic of patient safety interventions in FCV settings (i.e. "patient safety" and "intervention"). Search terms related to setting will be developed using the annually updated List of Fragile and Conflict-affected Situations as defined by the World Bank Country and Lending Groups from 2011-2021 [13]. Databases searched will include PubMed/Medline and Embase, as well as grey literature, particularly the outputs of non-

For peer review only - http://bmjopen.bmj.com/site/about/guidelines.xhtml

governmental organisations (NGOs). Papers published between 2011 and 2021, in English language, will be included.

The experts who will participate in the subsequent eDelphi will be asked to answer an exploratory question (free text) to list as many interventions as they want or a set number of responses to the question: 'Which interventions are needed to improve patient safety in FCV settings?'. The questionnaire will be hosted on Qualtrics, with a link to the survey distributed via email to all participants followed by two reminder emails, at one-week intervals.

Data collection

Qualtrics will be used to develop an online survey for each round of the eDelphi. The survey will be conducted across three rounds and each one will take approximately 15 minutes or less to complete. A link to each survey will be distributed via email to all participants followed by two reminder emails, at one-week intervals, per survey round.

In round 1, participant demographics will also be collected, including: gender, year of birth, country of residence, and current professional role. In Rounds 1-2, experts will be asked to rank the interventions by answering the question: "This intervention is relevant to improve patient safety in FCV settings". Responses will be captured in a four-point Likert scale ('strongly agree', 'agree', 'disagree', 'strongly disagree'). The four-point Likert scale has been utilised in previous Delphi studies, producing stable findings [14]. For each statement, participants will be given the option to select 'don't know' as an alternative response. A free-text response will also be made available to participants after each statement, facilitating them to elaborate on their thoughts or explain their responses. In Round 3, experts will be presented with the individual and group results from Round 2, to enable them to reconsider their responses in this round. With this information, participants will be asked to order each of the interventions that received consensus in Rounds 1 and 2 based on which are likely to be most impactful. Responses will be subsequently analysed and ultimately mapped in thematic areas ranked by importance.

Expert panel recruitment

For peer review only - http://bmjopen.bmj.com/site/about/guidelines.xhtml

Delphi studies employ a panel of expert members, as opposed to a random representative sample. In this study, ‘expert’ will be defined as a professional with experience working in health delivery and/or patient safety in FCV settings and experts working on this topic area. For the purposes of recruitment in the study, “FCV settings” will be considered as per the definition of WHO (i.e. settings experiencing humanitarian crises, protracted emergencies, prolonged disruption to critical public services or governance (e.g., due to political or economic challenges, conflict or natural disaster), or armed conflict”.[4] These settings can be entire countries (e.g., Syria) where war has affected the national health system, or specific geographic areas of countries that have been affected (e.g., the aftermath of a natural disaster, an influx of refugees to a particular area, etc.) This definition enables the recruitment of experts across high, middle- and low-income health systems to gather a broad range of opinions (see Figure 1). We will recruit experts on the countries listed in the World Bank FY22 List of Fragile and Conflict-affected Situations, as well as individuals working in these settings [5]. We will also recruit experts who currently work, or have previously worked, in settings at the intersection outlined in Figure 1. Participants will be included if aged 18 years old or older and able to understand English.

[Figure 1 around here]

Figure 1: Conceptual framework for expert panel recruitment

Purposive sampling will be used to ensure representativeness from a variety of expert roles (clinicians, managers/administrators, policymakers, researchers, and NGOs) at both organisational and governmental level. The majority of participants will be established contacts of the research team via the Institute of Global Health Innovation’s (IGHI) Leading Health Systems Network (LHSN) and the NIHR Imperial Patient Safety Translational Research Centre (PSTRC). Other relevant participants will be identified through the team’s contacts at the World Health Organization (WHO) or using snowball sampling. Attention will be paid to ensure the range of participants is geographically diverse and reflects the wide range of FCV settings, including settings of current or recent armed conflict, complex emergencies, natural disasters, and disruption to public services.

There is no agreement in the literature on the optimal number of participants to include in a Delphi survey [15]. Therefore, we will include all ‘experts’ who express an initial interest to participate.

For peer review only - http://bmjopen.bmj.com/site/about/guidelines.xhtml

An initial invitation email will be sent to possible participants identified by the research team. Participants will be made aware that their involvement is voluntary, and if they do not take part in the study, it will not affect their relationship with the study team. Each participant will be given a Participant Information Sheet and provided with the opportunity to address any queries they may have with a researcher.

Data analysis

Descriptive statistics will be used to describe participants' characteristics. The answers to the pre-Delphi question 'Which interventions are needed to improve patient safety in FCV settings?' (free text) will be analysed by two independent qualitative researchers using thematic analysis (NVivo Software). To cross-check data analysis and ensure data quality, consistency in approach, and transparency of analytical decision making, 50% of the data will be read and coded independently by a second experienced qualitative researcher [16]. Discrepancies in interpretation will be reviewed and resolved by a third qualitative researcher. A number of statements representing each theme will then be developed.

The answers to the Delphi rounds (i.e. ranking safety interventions as outlined) will be described using mean, median, standard deviation (SD), and interquartile range (IQR). Consensus will be defined as > 70% of participants agreeing/strongly agreeing or disagreeing/strongly disagreeing with a statement. This level of agreement has been previously considered appropriate in similar Delphi studies [17][18]. "Don't know" answers will be excluded from the analysis.

Patient and public involvement

Patient partners will be included in the interpretation of our results, in the co-development of a dissemination strategy, and in summarising the research findings into lay summaries and reports, in order to raise awareness and stimulate public participation on this topic. The research team at the Imperial NIHR PSTRC has a dedicated PPIE lead and a Research Partners Group.

Ethics and dissemination

For peer review only - http://bmjopen.bmj.com/site/about/guidelines.xhtml

This project has received ethics approval from the Imperial College London Ethics Committee, reference number 20IC665. Consent has been incorporated into the survey alongside the participant information provided. The results of this study will be published in a peer-reviewed journal and disseminated at national and international conferences.

Discussion

The aim of the study is to identify the most relevant patient safety interventions for FCV settings through an eDelphi research approach. Recognising the increasing scope of FCV settings, and their wide-ranging nature and impact on increasing numbers of the global population, we will identify critical actions to be taken forward.

Strengths and Limitations

There are both strengths and limitations for this study. The qualitative aspect of the study will enable the collection of detailed information from participants to answer the research question, and in addition may include the collation of relevant information that may not have been anticipated by the researchers [19]. It also offers the opportunity for the research team to explore participants' perceptions and the justifications for their responses in detail [20]. The use of a Delphi method specifically offers flexibility to adapt the research to reflect the unique and challenging topic, offers anonymity to the participants, and the opportunity to probe and develop existing knowledge and areas of controversy collectively [21, 22].

The nature of the eDelphi and the sampling strategy proposed will also enable the contribution of geographically diverse participants from regions around the world, and a good representation of those working in (and with lived experience) of these settings. The online nature of the eDelphi is further valuable in the context of the ongoing COVID-19 pandemic, as participation in the consensus building exercise will not be restricted by social distancing. Contributions will remain anonymous and will not be individually identifiable.

There are also several limitations to this work (see Appendix). Common critiques of the Delphi method tend to focus on the challenge of defining consensus, which can vary substantially from one Delphi research project to another, and the attrition rate of participants, which typically

For peer review only - http://bmjopen.bmj.com/site/about/guidelines.xhtml

increases in each round of the study [23]. In conducting research in FCV settings, we would expect attrition rates to be particularly high due to the nature of work and conflicting priorities among participants. We will seek to mitigate this challenge by addressing the time commitment required from participants, including reducing the length of the surveys to the minimum. Similarly, in the first instance we will seek a larger sample size to account for attrition.

The Delphi method has also been criticised for the role of response anonymity and the subsequent lack of participant accountability and ownership of ideas, which is further exacerbated in the eDelphi technique as physical anonymity is an additional characteristic [21,22,23]. Further, the fact that participants will not be in the same room to discuss their experiences and debate differences may lead to lack of engagement with the process. We will be mindful of this challenge and address any participant concerns through the course of the research. The nature of the eDelphi, specifically the use of online platform Qualtrics to administer the survey questionnaire instead of a traditional face-to-face approach, will require a stable internet source and internet access. Given the internet access and connectivity challenges in some of the most challenging FCV settings, some potential participants in the survey may be excluded from participating in the research. In the Appendix document, we outline several mitigation strategies noted by Donohoe et al. (2012) that may be relevant in the FCV content [22].

Finally, the eDelphi has been criticised for the inherent loss of research control as the research is moved online, giving the research team less oversight on appropriate participation, including ensuring expert participation and adherence to protocols [22]. As highlighted (see Appendix), the development of a risk register may offer the research team a lens by which to assess the major threats to research control in the FCV setting, including but not limited to, FCV-based disruptions and security challenges.

Implications for research and policy

This paper outlines the design of an eDelphi study to address the lack of research and evidence base for patient safety interventions in FCV settings. The aim of the study is to seek an understanding and gain consensus on the interventions that may be most relevant in FCV settings. Recognising the increasing scope of FCV settings, this work aims to identify critical actions to be

For peer review only - http://bmjopen.bmj.com/site/about/guidelines.xhtml

taken forward. This goal is aligned with the UHC agenda, which advocates for healthcare for all and prioritises the reduction of inequities in access to provision of care - and, ultimately, in safety outcomes.

The results of this study will create a list of the most relevant patient safety interventions, based on the consensus reached among a range of experts. The outcomes of this study have the potential to increase awareness in this area, and to identify interventions with a higher priority for implementation, as well as further evaluation and research.

Author contributions

ALN, AS and NO conceptualised the manuscript. ALN and AS planned the development of the work and ALN, AS and NO led in the writing of the original draft manuscript, with input from KF, SL, MD, and AD. All authors (AS, NO, KF, SL, MD, AD, ALN) equally contributed to the writing, reviewing, and editing to develop the final draft. All authors approved the version submitted for publication.

Funding statement

ALN is supported by the National Institute for Health Research (NIHR) Imperial Patient Safety Translation Research Centre (Award reference is PSTRC_2016_004). Infrastructure support was provided by the NIHR Imperial Biomedical Research Centre (BRC).

Competing interests

None declared.

Data sharing statement

Not applicable

For peer review only - http://bmjopen.bmj.com/site/about/guidelines.xhtml

Patient consent

Not required.

Disclaimer

The views of the authors do not necessarily reflect those of the NHS, NIHR or the Department of Health.

Provenance and peer review

Not commissioned; externally peer reviewed.

Acknowledgements

This work has not been previously published.

For peer review only - http://bmjopen.bmj.com/site/about/guidelines.xhtml

For peer review only

References

1. National Academies of Sciences Engineering and Medicine (NASEM). Crossing the Global Quality Chasm: Improving Health Care Worldwide. 2018. Washington DC. Available from: doi:10.17226/25152
2. Rice H E, Lou-Meda R, Saxton AT, Johnston BE, Ramirez CC, Mendez S, et al. Building a safety culture in global health: lessons from Guatemala. BMJ Global Health. 2018;3.
3. Syed S B, Leatherman S, Neilson M, Griekspoor A, Horemans D, Letaief M, et al. Improving quality of care in fragile, conflict-affected and vulnerable settings. Bull World Health Organ. 2020 Jan 1;98(1):2-2A.
4. Quality of care in fragile, conflict-affected and vulnerable settings: taking action. Geneva: World Health Organization; 2020.
5. World Bank. Classification of Fragile and Conflict-Affected Situations [Internet]. World Bank; 2021. Available from: https://www.worldbank.org/en/topic/fragilityconflictviolence/brief/harmonized-list-of-fragile-situations [cited 15 Jul 21].
6. Leatherman S, Tawfik L, Jaff D, Jaworski G, Neilson M, Letaief M et al. Quality healthcare in extreme adversity: Developing a framework for action. International Journal for Quality in Health care. 2020; 32 (2), 149-155.
7. WHO | WHO Surgical Safety Checklist [Internet]. WHO. World Health Organization. Available from: http://www.who.int/patientsafety/topics/safe-surgery/checklist/en/ [cited 2021 Apr 21].
8. Haynes A B, Weiser T G, Berry W R, Lipsitz S R, Breizat A-HS, Dellinger E P, et al. A Surgical Safety Checklist to Reduce Morbidity and Mortality in a Global Population. New England Journal of Medicine. 2009 Jan 29;360(5):491–9.
9. Weiser TG, Haynes AB. Ten years of the Surgical Safety Checklist. Br J Surg. 2018 Jul;105(8):927-929.
10. Keeney S, Hasson F, McKenna H P. A critical review of the Delphi technique as a research methodology for nursing. Int J Nurs Stud 2001; 38:195–200.
11. Merriam-Webster. "Intervention." Merriam-Webster.com [Internet]. Merriam-Webster, 2021. Web. 15 Jul 2021. Available from: https://www.merriam-

For peer review only - http://bmjopen.bmj.com/site/about/guidelines.xhtml

webster.com/dictionary/intervention [cited 15 Jul 21].

12. Tricco AC, Langlois EV, Straus SE, editors. Rapid reviews to strengthen health policy and systems: a practical guide. Geneva: World Health Organization; 2017.
13. World Bank. Classification of Fragile and Conflict-Affected Situations 2006 - 2020 [Internet]. World Bank; 2020. Available from: https://thedocs.worldbank.org/en/doc/176001594407411053-0090022020/original/FCSListFY06toFY20.pdf
14. Akins RB, Tolson H, Cole BR. Stability of response characteristics of a Delphi panel: application of bootstrap data expansion. BMC Med Res Methodol. 2005;5:37.
15. Keeney S, Hasson F, McKenna H. Consulting the oracle: ten lessons from using the Delphi technique in nursing research. J Adv Nurs 2006; 53:205–12.
16. Braun V. & Clarke V. Using thematic analysis in psychology. Qualitative research in psychology 2006; 3:77–101.
17. Slade SC, Dionne CE, Underwood M, Buchbinder R. Standardised method for reporting exercise programmes: protocol for a modified Delphi study. BMJ Open. 2014;4:e006682.
18. Diamond IR, Grant RC, Feldman BM, Pencharz PB, Ling SC, Moore AM, et al. Defining consensus: a systematic review recommends methodologic criteria for reporting of Delphi studies. J Clin Epidemiol. 2014 Apr;67(4):401-9.
19. Pope C, Royen P van, Baker R. Qualitative methods in research on healthcare quality. BMJ Quality & Safety. 2002 Jun 1;11(2):148–52.
20. Fitzpatrick R, Boulton M. Qualitative methods for assessing healthcare. Quality in Health Care. 1994. 3: 107-113.
21. Fink-Hafer D, Dagen T, Doušak M, Novak M, Hafner-Fink M. Delphi Method: Strengths and Weaknesses. Metodološki zvezki. 2019;16 (2): 1-19. Available from: https://www.researchgate.net/publication/337570516_Delphi_Method_Strengths_and_Weaknesses [cited 2021 Apr 21].
22. Donohoe H, Stellefson M, Tennant B. Advantages and Limitations of the e-Delphi Technique, American Journal of Health Education. 2013; (1): 38-46.
23. Shariff N J. Utilizing the Delphi Survey Approach: A Review. J Nurs Care. 2015; 4 (3).

For peer review only - http://bmjopen.bmj.com/site/about/guidelines.xhtml

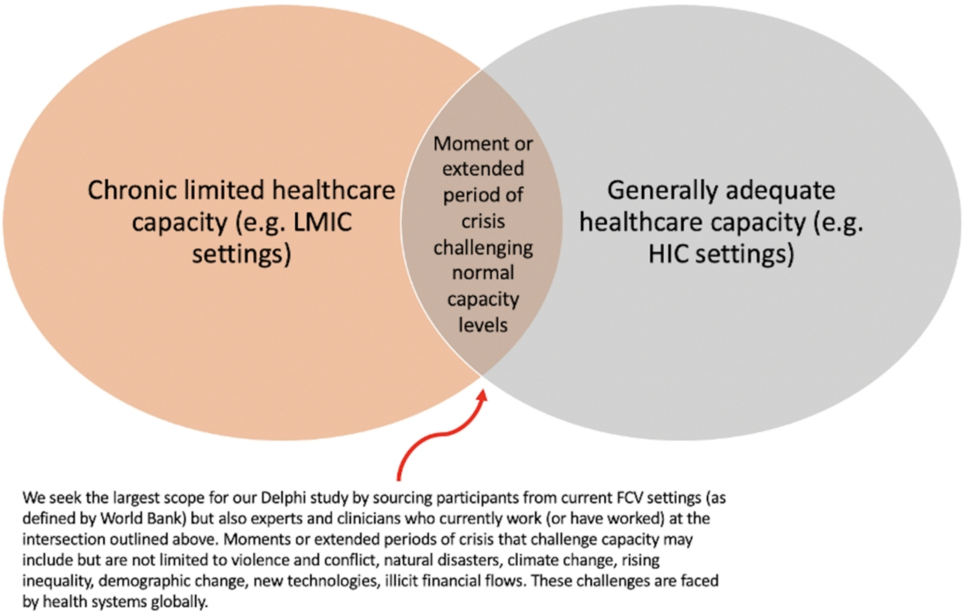

Figure 1: Conceptual framework for expert panel recruitment

165x106mm (300 x 300 DPI)

Appendix

Select Delphi study limitations	Reflections on limitations in the FCV research content	Mitigation strategies in the FCV research context
Challenge in defining consensus	No unique challenges in defining consensus in FCV research.	A clear plan to define consensus, based on best practice and in line with published research, agreed by the research team could be beneficial at the protocol development stage.
High attrition rates	Attrition rates in FCV research are likely to be higher than in other areas. Individuals involved in FCV research working in FCV settings will likely have several competing priorities due to a lack of healthcare resources in these settings.	Attrition rates should be considered in protocol development, with steps taken to reduce the number of rounds and length of questionnaires to capture only the information required for the study.
Anonymity and accountability	Anonymity may play a more complex role in FCV research as the information discussed by participants is likely to have an emotional component and may involve the sharing of negative experiences.	The research team should be mindful of the negative aspects of anonymity in Delphi studies and work with participants to ensure that the sharing of personal experiences by participants (and respective analysis) are undertaken in a respectful manner with particular attention to the emotive nature of the topic, and participant well-being.

For peer review only

Internet access	Internet access challenges for some participants in FCV settings is likely to be a challenge. In FCVs, connectivity may be poor or non-existent, thus excluding individuals in these settings from participating in the study.	Depending on the nature of the research project and funding available there are several mitigation strategies that could be employed. For example, Donohoe et al. (2012) suggest making paper surveys available to participants with no internet access / limited access or offering financial assistance to facilitate access where appropriate [1].

For peer review only

Loss of research control	In FCV settings, there are additional risks in losing research control, specifically missing deadlines due to a range of possible distractions and delays to participation. Additionally, the security of data related to individual participation may be more challenging in the FCV environment.	The research team could develop a risk register outlining risks to maintaining control. Additionally, Donohoe et al. (2012) note the value of identifying possible distractions/delays to participation [1]. In the FCV setting, FCV-based disruptions (e.g., political challenges, weather challenges) should also be considered.<br>Similarly, Donohoe et al. (2012) recommend ensuring secure survey access through unique passwords and hyperlinks [1]. In FCV settings, this may be increasingly important as systems may have lower cybersecurity protection in place and healthcare organisations may be a frequent the target of attacks (both physical and cyber) [2,3].

References

1. Donohoe H, Stellefson M, Tennant B. Advantages and Limitations of the e-Delphi Technique, American Journal of Health Education. 2013; (1): 38-46.
2. International Committee of the Red Cross. What limits does the law of war impose on cyber attacks? Available from: https://www.icrc.org/en/doc/resources/documents/faq/130628-cyber-warfare-q-and-a-eng.htm [cited 2021 April 21].

For peer review only

3. International Committee of the Red Cross. Call to governments: Work together to stop cyber attacks on health care. Available from: https://www.icrc.org/en/document/governments-work-together-stop-cyber-attacks-health-care [cited 2021 April 21].

For peer review only
